# Biomedical Insights That Inform the Diagnosis of ME/CFS

**DOI:** 10.3390/diagnostics10020092

**Published:** 2020-02-08

**Authors:** Brett A. Lidbury, Paul R. Fisher

**Affiliations:** 1 National Centre for Epidemiology and Population Health, Research School of Population Health, Australian National University, Canberra, ACT 2601, Australia; 2 Department of Physiology, Anatomy, and Microbiology, La Trobe University, Melbourne, VIC 3086, Australia; P.Fisher@latrobe.edu.au

It is well known that myalgic encephalomyelitis (ME) and chronic fatigue syndrome (CFS), whether considered as separate diseases or as the one chronic syndrome, continue to generate debate. Discussions on language, definitions and theoretical parameters continue, but whatever your position, one can now agree that ME and/or CFS (referred to hereafter as ME/CFS) is a disease with a physiological basis, rooted in biochemical and molecular dysfunction in the cells of sick individuals, and not attitudes that can be alleviated by psychological therapies. As a result, biomedical imperatives must now become the focus of research enquiry in order to find clinically translatable answers as soon as possible.

This book is intended as a landmark volume to mark this shift in thinking and to consolidate recent fundamental discoveries and biomedical insights as pathways towards tangible diagnostics, and eventual ME/CFS treatments. Australian researchers, with their collaborators locally and abroad, have been at the forefront of discovery in the biomedical realm, and this book draws together fundamental and applied insights that have emerged from scientific and clinical enquiry. The consolidation of up-to-date insights into ME/CFS was catalysed by a conference (https://www.emerge.org.au/symposium#.XbIv2iVS-3c) in March 2019 (Geelong, Australia), hosted by Emerge Australia, and several chapters in this volume are based on presentations from this meeting.

## Section I—Reviews, Commentaries and Opinions

The book is arranged into two sections, with the first presenting the up-to-date commentaries and reviews of the broader ME/CFS literature.

Cortes Rivera et al. [1] provide a “comprehensive review” of the field that takes us from some of the early recorded outbreaks, attempts to recognise the aetiology of disease, a summary of ME/CFS clinical and population features, and impacts on contemporary biomedical thinking. The expertise of some authors was reflected also by valuable commentary on the genetic basis of ME/CFS, including epigenetic studies, with interesting lessons for complex disease in general. Central to the book’s theme on “Biomedical Insights ….” are discoveries concerning ME/CFS patho-mechanism. Missailidis et al. [2] provides the required update on ME/CFS pathology as explained at the cellular level via biochemical and molecular alterations to function. Considering the array of triggers/causes of ME/CFS, but a similar range of symptoms, they suggest that patient stratification along these definitions is essential to understanding the molecular links to the whole person’s clinical picture.

The commentary by Sweetman et al. [3] nicely follows the general reviews, and squarely addresses the question of whether biomedical science has assisted the clinic, with their views on the biomedical potential of fundamental discoveries augmented by the presentation of data from their own research laboratory. The answer, not surprisingly, is yes and no. However, they venture opinions on the frustrations experienced by both patients and clinicians due to “… a long period of debate among the health profession about the true nature of the illness. It has hindered funding for much needed research and has created inertia for researchers to join the research effort”, while acknowledging the valuable discoveries of recent years, and the potential for progress in the near future. Such views are important to express, as they give impetus to a focused research effort, in spite of ongoing debate. Capturing an element of the debate, Twisk helpfully highlights a range of issues when considering ME as compared to the ME-ICC definitions (2011 International Consensus Criteria) [4]. In Twisk’s view, the ICC has helpfully abandoned the “CFS” nomenclature, but continuing confusion exists since ME and ME-ICC definitions have not been harmonised.

To complete the review and commentary section of the book, Vink and Vink-Niese [5] review the situation pertaining to ME/CFS impairment and disability in relation to work, with a focus on diagnostic strategies and prognosis, as measured by the style and intensity of work possible post diagnosis. Of particular value is that the article draws upon the experience of one author (Vink), who is an occupational physician expert in the evaluation of patient disability, and discusses how to best facilitate a return to the workplace. To our knowledge, this is the first occasion that a specific examination of ME/CFS and its occupational impact has been published. Not only is the evidence reviewed, but advice is provided on how best to prepare ME/CFS patients for a return to work, if at all possible.

## Section II—Research Results—Biomedical Insights and Diagnostics

The following section will highlight results obtained from primary biomedical research inquiry, and as such, represents contemporary thinking on the mechanisms of disease. For complex diseases like ME/CFS, research from diverse perspectives is essential. Section II starts with a qualitative study based on surveys and clinical observations, moving to new ways of using pathology laboratory data to provide marker patterns to assist diagnosis (as well as understand aetiology), with the final chapters highlighting exciting developments at the metabolomic and cellular levels of function.

Holtzman et al. [6] developed a survey, with community collaboration, to specifically assess PEM (post-exertional malaise). The surveys took the form of self-report questionnaires that were subsequently completed by over 1500 members of the patient community (35 countries, 41.1% from the USA). In the opinions of consulted community members, the most valuable PEM domains included onset triggers, timing and duration, the contribution of “personal characteristics”, among other factors not previously investigated. The authors also proposed their study as a model of community collaboration, with valuable outcomes for patients, while declaring that they lacked knowledge of what case definitions were applied to individuals in the study cohort, and did not seek independent evaluation. As alluded to earlier, the variety of case definitions/criteria continues to bedevil progress on ME/CFS.

For patients who fulfil the diagnostic clinical criteria for ME/CFS, a feature of laboratory (pathology) tests is that all results across blood and biochemical markers report within the analyte reference intervals, suggesting no physiological dysfunction (but remain useful for excluding other health conditions). Nacul et al. [7] have confirmed this observation in the records of UK ME/CFS Biobank (UKMEB) participants, but found that for a normally non-requested blood test marker, creatine kinase (CK), severe cases had a significantly (*p* < 0.001) reduced serum concentration compared to healthy controls, with fluctuations in CK concentrations associated with symptom severity. Serum CK concentration variation persisted despite correction for disease duration, age, sex, and so on, encouraging further investigation of CK as a diagnostic marker.

The absence of pathology test results outside of the reference intervals was reported also by Lidbury et al. [8] for an Australian cohort recruited from the Melbourne region. For this investigation, the machine learning (ML) algorithm random forest (RF) was applied to identify predictor patterns from the pathology results, both for the direct comparison of ME/CFS to healthy control participants, as well as via the weighted standing time (WST) proxy for symptom severity. Serum urea and 24-hour urinary creatinine, markers of nitrogen metabolism, were found as the leading markers to differentiate ME/CFS from health, as well as degree of symptom severity. The role of the cytokine activin B as a serum marker was further examined along with the range of pathology tests, and was found to be significantly reduced in the serum of ME/CFS patients, in addition to being useful in differentiating moderate to severe symptom severity when added to RF models.

The identification of nitrogen markers within pathology test results link to deeper metabolomic analyses in samples from ME/CFS patients, as demonstrated by previous results from Gooley, Armstrong, and McGregor, who reported abnormalities in urea cycle metabolites. Further work by McGregor et al. [9] is presented here, which focused on biochemical alterations during self-reported PEM episodes. Glycolytic anomalies were indicated by glucose:lactate ratios, which correlated with a fall in the purine metabolite hypoxanthine. A “hypermetabolic event” was suggested by increases in the urinary excretion of methyl-histidine, mannitol, and acetate. In addition to these observations, data indicated a role for hypoacetylation, showing that multiple biochemical events from histone function to physical gut and muscle symptoms coincide with PEM.

The metabolomic biochemistry theme is further explored by Kashi et al. [10], who propose the indolamine-2,3-dioxygenase (IDO) metabolic trap hypothesis. The hypothesis explores the link of IDO biochemistry in the context of kynurenine pathways, and the amino acid transporter LAT1, through mathematical models of tryptophan metabolism. The formulation of the IDO hypothesis eventuated from understanding the history of outbreaks world-wide, database searches for common “damaging” mutations in human enzymes, and the synthesis of the hypothetical implications for ME/CFS through mathematical models. For example, the balance of tryptophan “steady-state” as physiological or pathological outcomes is presented, with the authors extending into experimental designs to test their hypothesis. As a disease with a “trigger”, the disruption of steady-states, and thereafter perturbations in metabolism, these characteristics fit our understanding of ME/CFS aetiology.

## Concluding Remarks

To reiterate, this book, as a Special Issue of the MDPI journal *Diagnostics*, stands as a landmark to consolidate the extent and value of biomedical research into ME/CFS, and associated clinical observations. Another metaphor may be a “rallying point”, especially for those who have accepted the physiological basis of ME/CFS, but have been discouraged by ongoing disagreement among the research community and health professionals. In spite of debate, the evidence presented here and elsewhere provides sufficient impetus to explore ME/CFS as a biomedical challenge that can be solved.

Guided by the journal title, all contributors were encouraged to focus on elements within their research or practice that emphasised diagnostic utility and innovation. Having succeeded in presenting a collection of manuscripts spanning patient experience to pathology, physiology to molecular and cellular biology, we hope that this publication invites further insights from biomedical science, and finally acceptance that ME/CFS is a true disease with physiological foundations.
